# Levosimendan pretreatment improves survival of septic rats after partial hepatectomy and suppresses iNOS induction in cytokine-stimulated hepatocytes

**DOI:** 10.1038/s41598-019-48792-z

**Published:** 2019-09-16

**Authors:** Tatsuma Sakaguchi, Yuki Hashimoto, Hideyuki Matsushima, Hidehiko Hishikawa, Mikio Nishizawa, Tadayoshi Okumura, Masaki Kaibori

**Affiliations:** 1 Department of Surgery, Kansai Medical University, Hirakata, Osaka Japan; 2 Department of Biomedical Sciences, College of Life Sciences, Ritsumeikan University, Kusatsu, Shiga Japan; 3 Research Organization of Science and Technology, College of Life Sciences, Ritsumeikan University, Kusatsu, Shiga Japan

**Keywords:** Interleukins, Tumour-necrosis factors

## Abstract

We evaluated the survival effects and biochemical profiles of levosimendan in septic rats after partial hepatectomy and investigated its effects in cultured hepatocytes. Thirty-two rats underwent 70% hepatectomy and were randomised equally into four groups, followed by lipopolysaccharide (LPS) injection (250 µg/kg, i.v.) after 48 h. Levosimendan was given (i.p.) 1 h before LPS injection [group (A) levosimendan 2 mg/kg; (B) 1; (C) 0.5; (D) vehicle]. Survival at 7 days was increased significantly in group A compared with that in group D [A: 63%; B: 38%; C: 13%; D: 0%]. In serum, levosimendan decreased the level of tumour necrosis factor-α, interleukin (IL)-1β, IL-6 and nitric oxide (NO). In remnant livers, levosimendan inhibited inducible nitric oxide synthase (*iNOS*) gene expression. In primary cultured rat hepatocytes stimulated by IL-1β, levosimendan suppressed NO production by inhibiting iNOS promoter activity and stability of its mRNA.

## Introduction

Levosimendan is a calcium sensitiser licensed in numerous countries to treat decompensated heart failure^1^. It acts by: (i) increasing the sensitivity of troponin C to calcium in myocardial cells, leading to inotropy; (ii) opening mitochondrial adenosine triphosphate (ATP)-sensitive potassium channels in smooth muscle cells, resulting in vasodilation^2^. Moreover, it is known that levosimendan treatment leads to reduction in proinflammatory cytokines and apoptosis signaling pathways in patients with heart failure^3^. Furthermore, in experimental sepsis model induced by cecal ligation and puncture (CLP), levosimendan showed cardioprotective effects through preventing cardiac inflammation^4^. The composite actions of levosimendan as an inotrope and anti-inflammatory support raise the theoretical possibility that levosimendan may have a value as a treatment of sepsis^5^. In animal studies, accumulating evidences suggest that levosimendan may mitigate multiple organ injuries besides the heart in conditions of septic shock or ischemia–reperfusion^2,5,6^, including lung injury in CLP model^7–9^, renal failure in LPS-induced endotoxemia^10^ and liver injury in hepatic ischemia–reperfusion^11^. In terms of underlying mechanisms, levosimendan exerted anti-inflammatory effects through probably decreasing nitric oxide (NO) release in sepsis^2,12^. However, the inhibitory effect of levosimendan on proinflammatory cytokine production cannot be solely attributed to alterations in the NF-κB pathway^12^. In a few limited case series and trials have shown a beneficial potential of levosimendan on cardiac^13^, renal^14^, pulmonary^15^ and hepatic^16^ function in patients with sepsis. However, the survival and organ protective benefits of levosimendan in patients with septic shock were not demonstrated in one randomised controlled clinical trial^6^. The optimal indications and protocols of levosimendan for sepsis treatment have not been established.

Sepsis after major hepatectomy is a major issue. There is general agreement that 70% hepatectomy alone is not fatal in rodents^17^, but intravenous injection of a sub-lethal dose of lipopolysaccharide (LPS) 48 h after partial hepatectomy (‘PH/LPS’-model) is associated with high mortality^18–21^. Reduced phagocytic function of the reticuloendothelial system after hepatectomy is considered to enhance endotoxin sensitivity^22^. LPS has a direct effect on macrophages (or Kupffer cells) to activate nuclear factor (NF)-κB, which induces expression of proinflammatory cytokines and inducible nitric oxide synthase (iNOS). The latter produces an excess of NO, which has been implicated in tissue injury and assumed to be one of the triggers leading to septic shock and multiple-organ failure^23^.

Previously, we reported that fibronectin^18^, pirfenidone^19^, edaravone^20^ and sivelestat^21^ improved survival and prevented liver injury in PH/LPS-model rats. These agents commonly exerted survival benefits if they were administered before LPS injection and had inhibitory effects on iNOS induction in hepatocytes^24–26^. In primary cultured rat hepatocytes, interleukin (IL)-1β stimulates production of iNOS and NO markedly in the absence of other cytokines^27^, and prevention of expression of those proinflammatory mediators is a reliable indicator of liver protection^28^.

We hypothesised that levosimendan pretreatment improves the survival of PH/LPS-model rats by preventing the endotoxin-induced systemic inflammatory response and liver injury. In addition to experiments using the PH/LPS-model, we conducted analyses of IL-1β-stimulated primary cultured rat hepatocytes, as a simple *in vitro* model of liver injury, in the presence or absence of levosimendan for better understanding of the intracellular mechanisms involved.

## Results

### Effect of levosimendan pretreatment on survival of PH/LPS-model rats

**Figure 1:**
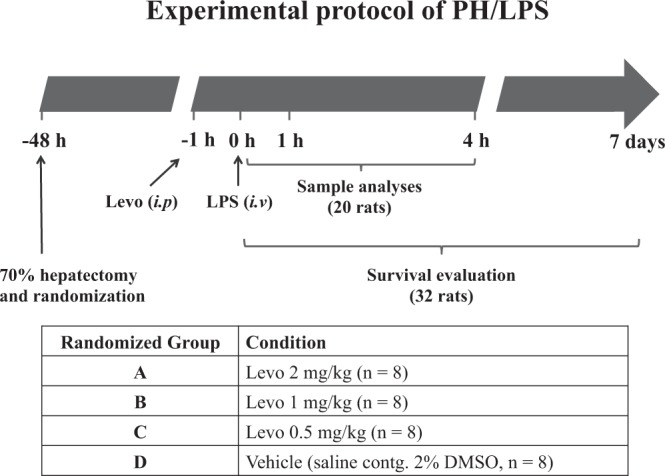
Experimental protocol of PH/LPS. Rats were treated with lipopolysaccharide (LPS, 250 µg/kg, i.v.) 48 h after 70% hepatectomy (PH/LPS). Levosimendan (Levo) or vehicle [saline containing 2% dimethyl sulfoxide (DMSO)] was administered (i.p.) 1 h before LPS injection. Survival of 32 rats was evaluated during 7 days. Samples from 20 rats were obtained at 0 h, 1 h or 4 h after LPS administration and analysed for an exploratory experiment.

**Figure 2:**
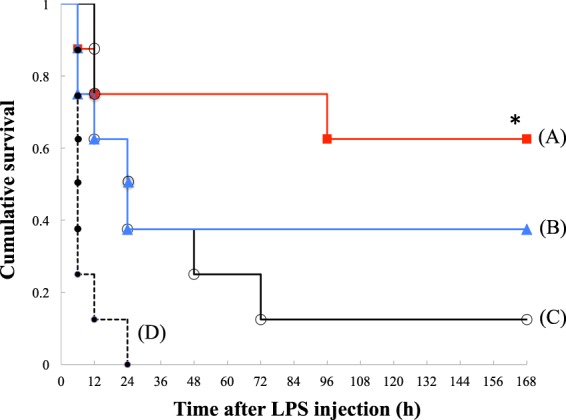
Effects of levosimendan on rat survival. Kaplan–Meier curves of PH/LPS are shown. (**A**) Levosimendan, 2 mg/kg, square; (**B**) 1 mg/kg, triangle; (**C**) 0.5 mg/kg, open circle; (**D**) vehicle, dot (8 rats per group). Each mark represents the death of rat in the indicated time. *P < 0.05 *vs*. (**D**).

A scheme of the experimental protocol of PH/LPS is shown in Fig. 1. Although we have less than 10% failure rate of PH/LPS model during the induction of anaesthesia and laparotomy, there was no rat to be lost during the interval between randomisation after laparotomy and LPS injection. Thirty-two operated rats were randomised equally into four groups and evaluated the survival during 7 days after LPS injection (Fig. 2). Rats administered vehicle (group D) began to die at 6 h and all rats died within 1 day after LPS injection. Survival of groups A, B and C at 7 days was 63%, 38% and 13%, respectively. The significant difference among four groups was confirmed (P < 0.01). According to post hoc analysis, survival of group A was significantly improved compared with group D (P < 0.01). A dose of 2 mg/kg was used in subsequent *in vivo* experiments.

### Effect of levosimendan on expression of cytokines, NO and transaminase in serum

**Figure 3:**
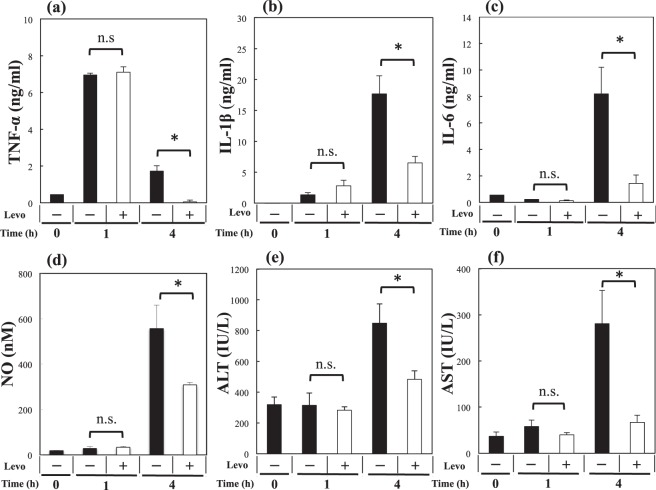
Effects of levosimendan on expression of cytokines, NO and transaminases in serum. Biochemical analyses of serum samples for **(a)** TNF-α, **(b)** IL-1β, **(c)** IL-6, **(d)** NO, **(e)** ALT and **(f)** AST are shown. Each graph consists of 5 bars representing 0 h (48 h after 70% hepatectomy without LPS or levosimendan treatment) as well as 1 h and 4 h after LPS treatment with levosimendan or vehicle. * and n.s. stand for *P* < 0.05 and not significant, respectively, between the shown pair.

The levels of tumour necrosis factor (TNF)-α, IL-1β, IL-6, NO, aspartate transferase (AST) and alanine transaminase (ALT) in serum were inhibited significantly (*P* < 0.01, 0.01, 0.02, 0.02, 0.04 and 0.02, respectively) by levosimendan at 4 h (Fig. 3a–f).

### Effect of levosimendan on expression of NF-κB, iNOS and cytokines in remnant livers

**Figure 4:**
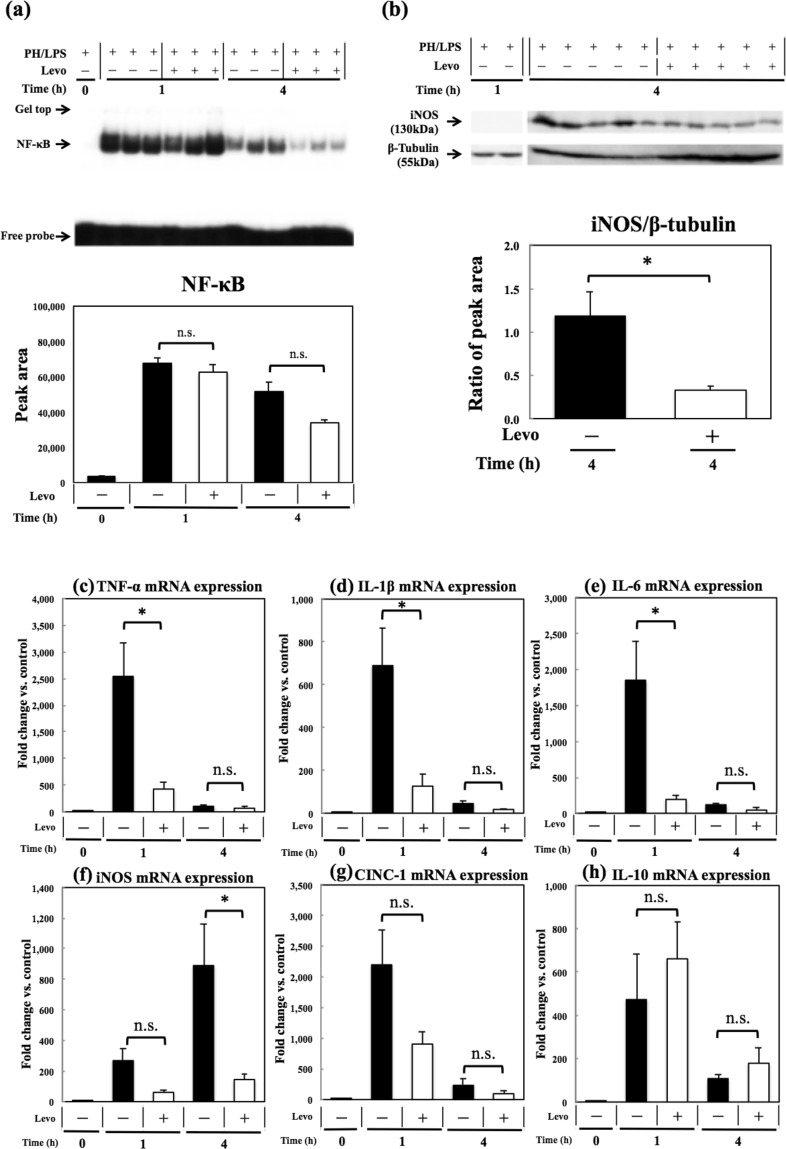
Effects of levosimendan on expression of NF-κB, iNOS and cytokines in livers. (**a**) The result of EMSA for remnant-liver samples is shown (upper), which consists of representatives of PH/LPS with vehicle at 0 h (3 rats), PH/LPS with vehicle at 1 h (3 rats), PH/LPS with levosimendan (Levo) at 1 h (4 rats), PH/LPS with vehicle at 4 h (4 rats) and PH/LPS with Levo at 4 h (5 rats). Full-length gels and full sample data are shown in a Supplementary information file. The density of blots in each group was quantified by densitometry (lower). **(b)** Results of western blotting for remnant-liver samples using primary antibodies against iNOS (upper) and β-tubulin (lower) at 1 h (representative 2 samples) and 4 h (5 samples in each group) after LPS treatment with levosimendan or vehicle are shown (Full-length gels are shown in a Supplementary Information file). **(c-h)** RT-PCR results for **(c)** TNF-α, **(d)** IL-1β, **(e)** IL-6, **(f)** iNOS, **(g)** CINC-1 and **(h)** IL-10. Each graph consists of five bars representing 0 h (48 h after 70% hepatectomy without LPS or levosimendan treatment), 1 h and 4 h after LPS treatment with levosimendan or vehicle. * and n.s. stand for *P* < 0.05 and not significant, respectively, between the shown pair.

The level of NF-κB in remnant liver was activated by LPS injection at 1 h, and then attenuated at 4 h, after LPS injection was examined by electrophoretic mobility shift assays (EMSAs). Activation of NF-κB tended to be decreased by levosimendan at 4 h after LPS injection, without significant differences (*P* = 0.056) (Fig. 4a). iNOS expression in remnant livers was inhibited significantly (*P* = 0.01) by levosimendan at 4 h (Fig. 4b). Levosimendan inhibited expression of the mRNA of TNF-α, IL-1β and IL-6 significantly (*P* = 0.03, 0.03 and 0.03, respectively) at 1 h, but insignificantly (*P* = 0.8, 0.09 and 0.15, respectively) at 4 h (Fig. 4c–f). iNOS mRNA was inhibited significantly (*P* = 0.03) at 4 h, but insignificantly (*P* = 0.1) at 1 h. Expression of cytokine-induced neutrophil chemoattractant (CINC)-1 mRNA tended to be inhibited at both 1 h and 4 h, without significant differences (P = 0.1 and 0.2) (Fig. 4g). Expression of IL-10 mRNA tended to be increased at both 1 h and 4 h, without significant differences (P = 0.6 and 0.4) (Fig. 4h).

### Effect of levosimendan on histopathological changes

**Figure 5:**
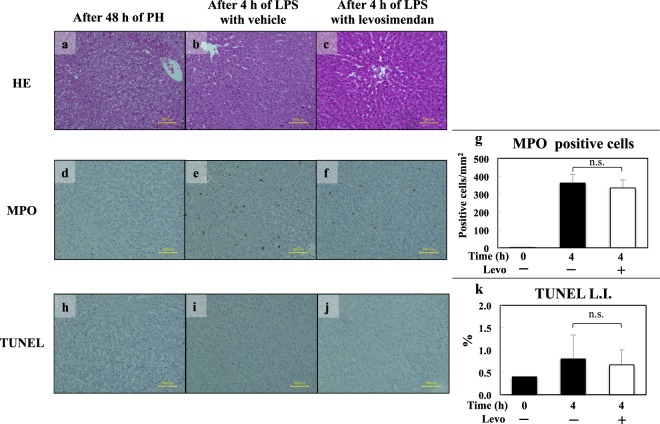
Effects of levosimendan on liver histopathology. Histopathology of remnant liver specimens of H&E staining (**a**–**c**), MPO staining (**d**–**f**) and TUNEL staining (**h**–**j**) are shown. Figure (**a,d,h)** are specimens of rats after 48 h of PH; figure (**b,e,i)** are rats after 4 h of LPS injection with vehicle; figure (**c,f,j)** are rats after 4 h of LPS injection with levosimendan. Graph (**g)** shows the result of MPO-positive cell counts (per mm^2^) in each group. Graph (**h)** shows the result of TUNEL-positive cell counts (per mm^2^) in each group. Each figure is a representative of each condition, and data of each graph represent the mean ± SE (n = 3–5 specimens/group). N.s. stands for not significant. Bar = 100 microns.

Histopathology revealed the change in regeneration of rat livers 48 h after 70% hepatectomy: ballooning hepatocytes and spreading of lipid droplets (Fig. 5a). After 4 h of LPS injection, focal necrotic hepatocytes were prominent at the centrilobular zone and midzone in both groups of PH/LPS with vehicle and levosimendan (Fig. 5b,c). Few myeloperoxidase (MPO)-positive cells were infiltrated in livers 48 h after 70% hepatectomy: (0.3 cells/mm^2^, Fig. 5d). Severe infiltration of MPO-positive cells was recognized in specimens of rat livers after 4 h of LPS injection with vehicle (Fig. 5e). Levosimendan pretreatment did not inhibit the infiltration of MPO-positive cells significantly in remnant livers (P = 0.7) (Fig. 5f,g). Apoptotic bodies were evaluated by terminal deoxynucleotidyl transferase-mediated dUTP-digoxigenin nick-end labelling (TUNEL) staining, and few positive nuclei were detected in rats 48 h after 70% hepatectomy (4 per 1,000 nuclei, Fig. 5h). The difference in the percentage of TUNEL-positive cells in all nuclei was not significant in the absence (Fig. 5i) or presence of levosimendan pretreatment (P = 0.9) (Fig. 5j,k).

### Effect of levosimendan on induction of expression of NO, iNOS protein and iNOS mRNA in IL-1β-stimulated cultured hepatocytes

**Figure 6:**
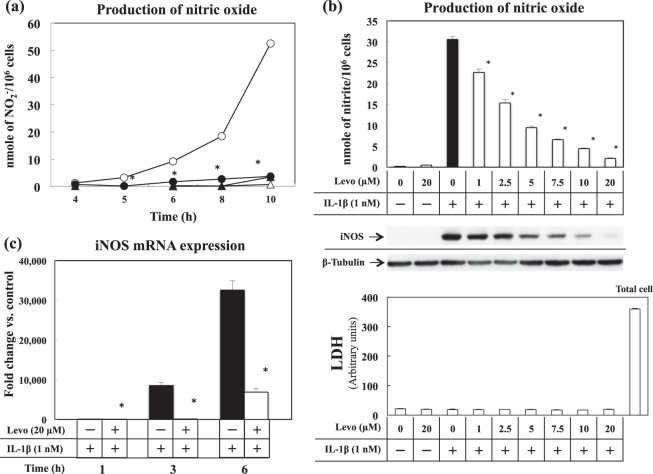
Effects of levosimendan on NO and iNOS induction in IL-1β-stimulated primary cultured hepatocytes. (**a**) Effects of levosimendan (Levo, 20 µM) on NO production for the indicated times (IL-1β only, open circles ○; IL-1β and Levo, closed circles ●; Levo only, closed triangles ▲; control, open triangles Δ). **(b)** Effects of Levo (1–20 µM) for 8 h on NO production (upper), iNOS and β-tubulin levels (middle, full-length gels are shown in a Supplementary Information file), and LDH activity (lower). **(c)** Effects of Levo (20 µM) on expression of iNOS mRNA for the indicated times. *P < 0.05 *vs*. IL-1β alone. n = 3 dishes/point or indication.

In the culture medium, simultaneous administration of levosimendan (20 µM) with IL-1β (1 nM) reduced the level of nitrite (NO metabolite) time-dependently, which was increased by single administration of IL-1β (Fig. 6a). Levosimendan reduced the production of NO and iNOS protein dose-dependently, and decreased production to a near-basal level at a concentration of 20 µM (Fig. 6b, upper and middle). The level of lactate dehydrogenase (LDH) in the culture medium was not increased by ≤20 µM of levosimendan (Fig. 6b, lower). A dose of 20 µM was used in subsequent *in vitro* experiments. Reverse transcription-polymerase chain reaction (RT-PCR) revealed that levosimendan reduced expression of iNOS mRNA in each hour (Fig. 6c).

### Effect of levosimendan on the activity of iNOS promoters, iNOS antisense transcription and intranuclear level of NF-κB in IL-1β-stimulated hepatocytes

**Figure 7:**
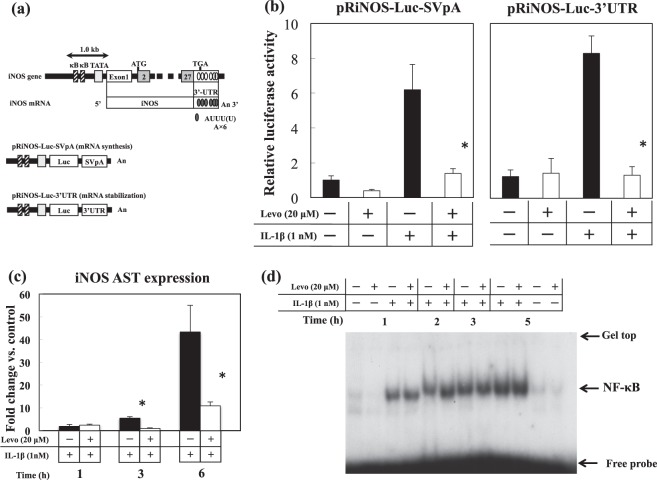
Effects of levosimendan on transactivation of *iNOS* promoters, *iNOS* AST expression, and binding of nuclear extracts to NF-κB consensus oligonucleotide. (**a**) Promoter region of *iNOS* (schematic). Two reporter constructs consisting of the rat iNOS promoter (1.0 kb), a luciferase gene, and the SV40 poly(A) region (pRiNOS-Luc-SVpA) or iNOS 3′-UTR (pRiNOS-Luc-3′UTR). “An” indicates the presence of a poly(A) tail. The iNOS 3′-UTR contains AREs (AUUU(U)A × 6), which contribute to mRNA stabilisation. (**b**) Relative luciferase activity of pRiNOS-Luc-SVpA and pRiNOS-Luc-3′UTR. *P < 0.05 *vs*. IL-1β alone (n = 6 dishes/indication). (**c**) Expression of iNOS AST for the indicated times. *P < 0.05 *vs*. IL-1β alone (n = 3 dishes/indication). (**d**) Nuclear extracts were analysed by EMSA (full-length gel is shown in a Supplementary Information file).

The scheme of the constructs containing firefly luciferase controlled by the iNOS promoter (pRiNOS-Luc-SVpA and pRiNOS-Luc-3′UTR) is shown in Fig. 7a. Levosimendan inhibited relative luciferase activities on both constructs, which were increased by IL-1β single administration (Fig. 7b). RT-PCR revealed that levosimendan inhibited expression of the iNOS antisense transcript at 3 h and 6 h (Fig. 7c). EMSAs with nuclear extracts did not show an inhibitory effect of levosimendan on NF-κB activation (Fig. 7d). Further, we could not detect significant influences of levosimendan on NF-κB nuclear translocation, IκB degradation and phosphorylation of NF-κB p65 (Ser^536^) (Supplementary information file; M, K, and L).

### Effect of levosimendan on the mRNA expression of pro-inflammatory cytokines in IL-1β-stimulated hepatocytes

**Figure 8:**
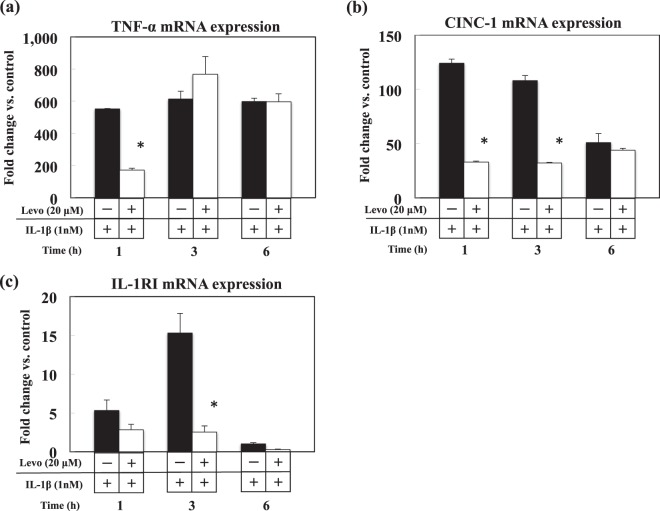
Effects of levosimendan on the mRNA expression of pro-inflammatory cytokines in IL-1β-stimulated hepatocytes. (**a**) TNF-α mRNA, (**b**) CINC-1 mRNA, and (**c**) IL-1RI mRNA. *P < 0.05 *vs*. IL-1β alone. n = 3 dishes/indication.

RT-PCR revealed that levosimendan suppressed expression of the mRNA of TNF-α, CINC-1 and the type-I IL-1 receptor (IL-1RI) at certain times (Fig. 8).

## Discussion

Two experimental models of sepsis with acute liver injury can be employed: (i) simultaneous administration of D-galactosamine/LPS^29–31^; (ii) PH/LPS. The lethal activity of endotoxins is enhanced considerably under both models, but the PH/LPS-model exhibits more severe and refractory symptoms^32^, and is closer to a specific clinical situation.

A pilot study revealed that a 50% lethal dose of LPS for this model was ≈100 µg/kg, and that >90% of rats died at a LPS dose of 250 µg/kg (i.v.) (T. O., unpublished observation). We chose the doses of levosimendan by reference to a similar study of ischemia–reperfusion injury in rat mesenteries^33^. In our preliminary study, administration of levosimendan (2 mg/kg) 1 h after LPS injection showed no effect on survival (data not shown). In contrast, pretreatment of levosimendan increased the survival of PH/LPS-model rats in a dose-dependent fashion, though a significant difference was only found between group A (doses of 2 mg/kg) and group D (vehicle) by post hoc analysis. Levosimendan pretreatment prevented an increase in expression of proinflammatory cytokines in serum and their mRNAs in remnant livers. Expression of iNOS in remnant livers and NO in serum (which are proinflammatory mediators) was also inhibited by levosimendan pretreatment. Those effects would probably involve inhibition of NF-κB activation, because NF-κB has an important role as a transcriptional factor of *iNOS* gene^28^. However, levosimendan did not inhibit NF-κB activation significantly shown in EMSA experiments in remnant livers. We should mention of the limited number of experimental animals we used and there probably existed the influence of other transcriptional factors such as hypoxia-inducible factor-1α^34^ or nuclear respiratory factor 2^35^. According to a reported study of septic mice, Wang *et al*. concluded that levosimendan did not inhibit the LPS-induced activation of NF-κB significantly, which is a similar result to our study^8^. Levosimendan demonstrated a hepatoprotective effect in that levels of transaminases in serum decreased significantly in the levosimendan group 4 h after LPS injection. However, histopathology revealed that levosimendan did not inhibit both the infiltration of MPO-positive cells (*i.e*., necrotic change) and TUNEL-positive cells (*i.e*., apoptotic change). The results of histopathology will cause controversy whether a hepatoprotective effect of levosimendan determined the survival benefit in our study. We assume that D-galactosamine/LPS-model would be essential for examining a heaptoprotective effect of levosimendan against LPS-induced acute liver injury^36^, but this model would not surely represent for septic shock^37^. As a limitation, we could not adopt a blinded maneuver of each group for a practical reason when we injected LPS and/or levosimendan. However, two researchers (T. S. and T. O.) assured the quality of experiments of PH/LPS.

In IL-1β-stimulated primary cultured hepatocytes, levosimendan suppressed NO production in a time- and dose-dependent fashion through inhibition of *iNOS* gene expression. We set the concentration of levosimendan at 20 μM in the experiments, because the levels of LDH in culture medium were slightly elevated at the concentration of 100 μM of levosimendan (data not shown), which implied cytotoxicity caused by the overdose of levosimendan, but levosimendan had no such effects at 1–20 μM. The experiments with iNOS promoter constructs demonstrated that levosimendan inhibited iNOS expression during the synthesis and stabilisation of mRNA. iNOS promoter activity measured with the constructs represented the intensity of NF-κB-dependent transcription because both constructs have two NF-κB binding sites (κB) in each promoter area. However, EMSAs revealed that the binding activity of nuclear extracts to the NF-κB consensus oligonucleotide was not inhibited by levosimendan. We conducted the additional experiments to investigate the NF-κB nuclear translocation, IκB degradation and phosphorylation of NF-κB p65 (Ser^536^), which are the important signalling steps to stimulate NF-κB activation. However, we could not detect significant influences of levosimendan on these steps (Supplementary file). From the results above, we concluded that levosimendan did not inhibit the activating steps of NF-κB in cultured hepatocytes. This result suggests that levosimendan might affect the synthesis of iNOS mRNA through signalling pathways and transcription factors other than NF-κB. We found that the iNOS antisense-transcript had a key role in stabilising iNOS mRNA by interacting with the 3′-ultratranslated region (UTR) and adenylate-uridylate-rich sequence elements-binding proteins^37^. Levosimendan demonstrated an inhibitory effect on expression of iNOS antisense transcripts. An anti-inflammatory profile of levosimendan was also shown in hepatocytes because of inhibition of the mRNA expression of TNF-α, CINC-1 and IL-1RI. Note that our *in vitro* study was not a complete reproduction of PH/LPS-model in two points that we did not use the direct cultured hepatocytes from all groups in PH/LPS-model, and we used a single cytokine (IL-1β) to stimulate the hepatocytes. The results from our *in vitro* study should be considered as reference to understand the anti-inflammatory mechanism of levosimendan.

Some *in vitro* studies have shown that levosimendan can down-regulate iNOS induction and NO production in response to inflammatory stimuli in macrophages^12^, cardiac fibroblasts^38^ and hepatocytes^39^. Differences in the signalling events leading to activation of iNOS transcription between cell types might exist. Sareila *et al*. reported that levosimendan did not affect the activation, nuclear translocation or DNA binding of NF-κB in J774 macrophages, but inhibited NF-κB-dependent transcription in L929 fibroblasts^12^. Okada *et al*. reported that levosimendan inhibited IL-1β-induced apoptosis *via* activation of the phosphatidylinositol-4, 5-bisphosphate 3-kinase/Akt pathway in the cardiac fibroblasts of adult rats^38^. The data from those studies are similar to our results.

As an *in vivo* model of sepsis, CLP-model^7,8^ has previously been used to show a survival benefit of levosimendan. Authors selected continuous infusion *via* a catheter in the jugular vein^7^ or an intraperitoneal osmotic pump^8^ of levosimendan, whereas we used intraperitoneal bolus administration. One may argue that intraperitoneal bolus administration of levosimendan does not represent the clinical situation accurately. However, the sepsis model caused by LPS injection does not fully represent human sepsis because LPS causes a much earlier peak of expression of pro-inflammatory cytokines compared with that seen in human sepsis. A survival curve of PH/LPS model is more precipitous that the majority of positive control rats died at 6 h after LPS injection compared with CLP-model that two-thirds of controlled rats survived at 9 h after operation^7^. Levosimendan has a half-life of ≈1 h but its active metabolite, OR-1896, has a half-life of 80 h^40^, which could cover the duration of effect of a LPS bolus administration. Continuous infusion of levosimendan in the PH/LPS-model may merit further study.

As *in vivo* model of liver injury, Grossini *et al*. reported that levosimendan protected against ischemia–reperfusion injury through mechanisms related to NO production and mitochondrial ATP-dependent potassium-channel function^11^. Taken together, levosimendan would have a beneficial effect in liver surgery/transplantation. The results of our study lead us to recommend levosimendan pretreatment for sepsis management after acute liver injury.

## Methods

### Animals

All animal experiments were undertaken in accordance with the *Guidelines for the care and use of laboratory animals* (National Institutes of Health, Bethesda, MD, USA). The study protocol was approved by the Animal Care Committee of Kansai Medical University (Permission numbers: 17-023(01) and 18-027(01)).

Rats (specific pathogen-free) were purchased from Charles River Laboratories Japan (Yokohama, Japan) and maintained in a room at 22 °C under a 12-h light–dark cycle with a diet of γ-irradiated CRF-1 (Oriental Bioservice, Kyoto, Japan) and water *ad libitum*.

### Drugs

Levosimendan was purchased from Wako Pure Chemical Industries (Osaka, Japan). Levosimendan was resolved in dimethyl sulfoxide (DMSO) and stored at −80 °C. For PH/LPS experiments, resolved levosimendan was diluted by 1 ml of normal saline for each rat so that the DMSO concentration was 2%. Isoflurane, pentobarbital sodium, collagenase, Transaminase CII-test kit, LDH-Cytotoxicity Assay kit and PicaGene Luminescence kit were from Wako Pure Chemical Industries. LPS (*Escherichia coli* O111:B4) and mouse anti-β-tubulin were from Sigma–Aldrich Japan (Tokyo, Japan).

Recombinant human IL-1β (2 × 10^7^ U/mg protein) was purchased from MyBioSource (San Diego, CA, USA). Enzyme-linked immunosorbent assay (ELISA) kits for TNF-α, IL-1β and IL-6 were from Life Technologies Japan (Tokyo, Japan). Rabbit anti-iNOS, TRIzol™ Reagent, and UltraPure™ DNase/RNase-Free Distilled Water were from Thermo Scientific (Waltham, MA, USA). ECL western blotting detection reagents were from GE Healthcare Japan (Tokyo, Japan). Luminate Forte Western Horseradish Peroxidase (HRP) was from Merck Japan (Tokyo, Japan). Oligo (dT) Primer (25 ng), 5 × RT Buffer, dNTPs Mixture, RNase Inhibitor and Rever Tra Ace^®^ were from Toyobo (Osaka, Japan). Magnet-assisted transfection (MATra) Reagent was from IBA (Gottingen, Germany). Beta-Glo kits, mouse immunoglobulin κ light chain was from Promega (Fitchburg, WI, USA). An *In situ* Apoptosis Detection kit (MK500) was from Takara Bio (Shiga, Japan). An Anti-myeloperoxidase rabbit polyclonal antibody (A0398) was from DAKO (Glostrup, Denmark).

### Creation of the PH/LPS model

The procedure for 70% partial hepatectomy is based on experiments described elsewhere^41^. Briefly, male Sprague–Dawley rats (8 weeks; 310 ± 10 g) were anaesthetised using pentobarbital sodium (40 mg/kg, i.p.) and isoflurane (0–2%). A laparotomy was done with a midline incision (≈3 cm). The left lateral and left median lobe of the liver were removed after ligation, followed by wound closure. Operated rats were randomised immediately and equally into four groups: (A) levosimendan 2 mg/kg; (B) levosimendan 1 mg/kg; (C) levosimendan 0.5 mg/kg; (D) vehicle (normal saline). Forty-eight hours after surgery, 250 µg/kg body weight of LPS in saline was injected into the penile vein. Levosimendan (i.p.) was given 1 h before LPS injection. Survival was evaluated during 7 days after LPS injection, and then rats were killed by isoflurane. As an exploratory experiment, samples of blood and remnant liver were taken from Sprague–Dawley rats 0 h, 1 h and 4 h after LPS injection with or without levosimendan pretreatment (n = 3–5 in each group). A scheme of the experimental protocol is shown in Fig. 1.

### Isolation and culture of primary hepatocytes

The isolation and culture of rat hepatocytes is based on experiments described elsewhere^42,43^. Hepatocytes were isolated from livers of Wister rats (200–220 g) by collagenase perfusion *via* the portal vein, followed by centrifugation (50 × g, 70 sec, 4 °C; four times). Isolated hepatocytes were suspended at 6 × 10^5^ cells/mL in Williams’ E (WE) culture medium, supplemented with 10% newborn calf serum, Hepes (5 mM), penicillin (100 U/mL), streptomycin (100 μg/mL), fungisone (0.25 μg/mL), aprotinin (0.1 μg/mL), dexamethasone (10 nM) and insulin (10 nM). The cells were seeded into 35- or 100-mm plastic dishes (2 or 10 mL/dish; Falcon Plastic, Oxnard, CA, USA) and cultured at 37°C in a CO_2_ incubator under a humidified atmosphere of 5% CO_2_ in air for 2 h. The medium (1.5 mL/35-mm dish) was replaced with fresh serum-free and hormone-containing WE medium (first medium change), then with fresh serum- and hormone-free WE medium at 5 h (second medium change), and the cells were cultured overnight. As cells were cultured two days or more before use in experiments, fresh serum-free and hormone-containing WE medium was used in the second medium change, with this medium subsequently changed every day. Then, cells were treated with recombinant human IL-1β (1 nM) in the presence or absence of levosimendan.

### Biochemical analyses

Serum levels of TNF-α, IL-1β and IL-6 were measured using commercial ELISA kits. The sum of nitrite and nitrate (stable metabolites of NO) in the serum, or nitrite in the culture medium, was measured using the Griess reagent method^44^. Serum levels of AST and ALT were determined using commercial kits. LDH activity in the culture medium was measured using a commercial kit according to manufacturer instructions.

### Western blotting

Protein extracts of liver sections and hepatocytes were prepared for western blotting, as described previously^29^. They were subjected to a 7.5% gel, and electroblotted. Immunostaining was done using primary antibodies against iNOS and β-tubulin (internal control), followed by visualisation with ECL Western Blotting Detection Reagents for iNOS and Luminate Forte Western HRP for β-tubulin. The bands corresponding to each protein were quantified by densitometry using ImageJ (San Diego, CA, USA)^45^.

### RT-PCR

Total RNAs of liver sections and hepatocytes were extracted in TRIzol Reagent using the guanidinium–phenol–chloroform method^46^. cDNA was synthesised from 1 µg of total RNA from each sample with Oligo(dT)20 Primer (25 ng), 5 × RT Buffer (5 µl), 10 mM of dNTPs Mixture (2.5 µl), RNase Inhibitor (0.5 µl), Rever Tra Ace (100 U) and UltraPure™ DNase/RNase-Free Distilled Water. The conditions of thermal cycling using iCycler (Bio-Rad Laboratories, Hercules, CA, USA) were 42 °C for 60 min and 95 °C for 5 min.

Real-time PCR was done using SYBR Green and primers for each gene. Primer sequences were synthesised by Eurofins Genomics (Tokyo, Japan) (Table 1). The conditions of thermal cycling using Rotor-Gene Q (Qiagen, Stanford, VA, USA) were 95 °C for 5 min followed by 40 cycles of 95 °C for 5 s and 60 °C for 10 s. Collection and analyses of data were done using the software included with the system. mRNA levels of each gene were measured as CT threshold levels and normalised to those of eukaryotic elongation factor-1α.

**Table 1:** Primer sets for RT-PCR Nucleotide sequences of primers.

Gene	RT Primer	PCR Forward Primer	PCR Reverse Primer	Amplification(bp)
EF-1α	oligo (dT)20	5′-TCTGGTTGGAATGGTGACAACATGC-3′	5′-CCAGGAAGAGCTTCACTCAAAGCTT-3′	332
iNOS	oligo (dT)20	5′-CCAACCTGCAGGTCTTCGATG-3′	5′-GTCGATGCACAACTGGGTGAAC-3′	257
TNF-α	oligo (dT)20	5′-TCCCAACAAGGAGGAGAAGTTCC-3′	5′-GGCAGCCTTGTCCCTTGAAGAGA-3′	275
IL-1β	oligo (dT)20	5′-TCTTTGAAGAAGAGCCCGTCCTC-3′	5′-GGATCCACACTCTCCAGCTGCA-3′	321
IL-6	oligo (dT)20	5′-GAGAAAAGAGTTGTGCAATGGCA-3′	5′-TGAGTCTTTTATCTCTTGTTTGAAG-3′	286
CINC-1	oligo (dT)20	5′-GCCAAGCCACAGGGGCGCCCGT-3′	5′-ACTTGGGGACACCCTTTAGCATC-3′	231
IL-10	oligo (dT)20	5′-GCAGGACTTTAAGGGTTACTTGG-3′	5′-CCTTTGTCTTGGAGCTTATTAAA-3′	245
IL-1RI	oligo (dT)20	5′-CGAAGACTATCAGTTTTTGGAAC-3′	5′-GTCTTTCCATCTGAAGCTTTTGG-3′	327
iNOS AST	5′-TGCCCCTCCCCCACATTCTCT-3′	5′-ACCAGGAGGCGCCATCCCGCTGC-3′	5′-CTTGATCAAACACTCATTTTATTAAA-3′	185

EF-1α, elongation factor-1-alpha; iNOS, inducible nitric oxide synthase; TNF-α, tumour necrosis factor-alpha; IL-1β, interleukin-1beta; IL-6, interleukin-6; CINC-1, cytokine-induced neutrophil chemoattractant-1; IL-10, interleukin-10; IL-1R1, type-I IL-1 receptor; iNOS AST, iNOS-antisense transcript.

### Transfection and luciferase assay

Transfection of cultured hepatocytes was undertaken as described previously^47^. Briefly, on day-0, hepatocytes were cultured for 7 h before being subjected to MATra. Reporter plasmid pRiNOS-Luc-SVpA or pRiNOS-Luc-3′UTR (1 µg) and the cytomegalovirus promoter-driven β-galactosidase plasmid pCMV-LacZ (1 ng) as an internal control were mixed with 1.5 µg of MATra-A reagent in 200 µL of Williams’ E medium. After incubation for 15 min on a magnetic plate, the medium was replaced and cultured overnight, and then treated with IL-1β in the presence or absence of levosimendan. Activities of luciferase and β-galactosidase in cell extracts were measured using PicaGene and Beta-Glo kits, respectively. Luciferase activity was normalised by β-galactosidase activity. Fold activation was calculated by dividing luciferase activity by control activity (without IL-1β and levosimendan).

### EMSA

EMSA was carried out as described previously^48,49^ with a minor modification, as described elsewhere^20,50^. Nuclear extracts were prepared from frozen liver at −80 °C or cultured hepatocytes. Binding reactions were undertaken by incubating the nuclear extracts in reaction buffer (20 mM of HEPES-KOH, pH 7.9; containing 1 mM of EDTA, 60 mM of KCl, 10% glycerol, and 1 µg of poly[dI-dC]) with a probe (40,000 dpm) for 20 min at room temperature. Products were electrophoresed on a 4.8% polyacrylamide gel in high-ionic-strength buffer, and dried gels were analysed by autoradiography. An NF-κB consensus oligonucleotide (5′-AGTTGAG GGGA-CTTTCCCAGGC) from the mouse immunoglobulin κ light chain was purchased and labelled with [γ-^32^P]-ATP and T4 polynucleotide kinase. Protein was measured using the Bradford method^51^. Bands corresponding to NF-κB were quantified by densitometry using ImageJ^45^.

### Histopathology

Specimens of remnant liver were fixed in 10% formalin solution and embedded in paraffin. Sections (3–5 µm) were cut and stained with haematoxylin and eosin. Neutrophil infiltration was evaluated by the counts of MPO-positive cells using the Anti-myeloperoxidase rabbit polyclonal antibody (A0398) per 20 HPFs under light microscopy. Apoptotic bodies were evaluated by TUNEL staining using the *In Situ* Apoptosis Detection kit (MK500) per 20 HPFs under light microscopy. The Labeling Index of TUNEL-positive cells per 1,000 hepatocyte nuclei was counted in duplicate.

### Statistical analyses

Comparison of rats’ survival among four groups was analysed statistically by one-way ANOVA, followed by Tukey-Kramer method (JMP^®^ 14, SAS Institute Inc., Cary, NC, USA). The results of *in vitro* studies in the figures are representative of at least three independent experiments that yielded similar findings. Data are the mean ± standard error (SE). Differences were analysed using the Student’s *t*-test and *P* < 0.05 was considered significant.
